# Clinical utility of early postoperative cardiac multidetector computed tomography after coronary artery bypass grafting

**DOI:** 10.1038/s41598-020-66176-6

**Published:** 2020-06-08

**Authors:** Doo Kyoung Kang, Sang Hyun Lim, Jin Sun Park, Joo Sung Sun, Taeyang Ha, Tae Hee Kim

**Affiliations:** 1 Department of Radiology, Ajou University School of Medicine, Suwon, Republic of Korea; 2 Department of Thoracic & Cardiovascular Surgery, Ajou University School of Medicine, Suwon, Republic of Korea; 3 Department of Cardiology, Ajou University School of Medicine, Suwon, Republic of Korea

**Keywords:** Outcomes research, Coronary artery disease and stable angina

## Abstract

We evaluated the clinical utility of early postoperative cardiac computed tomography (CT) for evaluating left ventricular (LV) function and predicting prognosis in patients who had undergone coronary artery bypass grafting (CABG). Of the 205 patients who underwent CABG from March 2011 to December 2014, 136 underwent early postoperative cardiac CT (within 30 days after CABG) and were enrolled as the study population. The baseline and postoperative follow-up echocardiographic findings, major adverse cardiac events (MACE), and death were recorded for a follow-up period (mean, 5.9 ± 1.1 years). Functional cardiac CT parameters were compared to echocardiographic measurements. The associations between cardiac CT findings and functional recovery and prognosis were evaluated by logistic regression analyses. The LVEF measured via cardiac CT was significantly higher (56.2 ± 11.5% vs. 61.9 ± 12.9%; p = 0.0002) compared to those via early postoperative echocardiography, but the wall motion score index (WMSI) was not significantly different (1.23 ± 0.33 vs. 1.21 ± 0.28, p = 0.5041) between the two methods. During the follow-up period, 17 patients (12.5%) died and 40 (29.4%) developed MACE. Both the LVEF and WMSI measured with early postoperative echocardiography (p = 0.0202 and odds ratio [OR] = 5.0171, p = 0.0039, respectively), and cardiac CT (OR = 0.9625, p = 0.0091 and OR = 14.3605, p = 0.0001, respectively) predicted MACE OR = 0.9630, but only the WMSI, measured using cardiac CT, predicted all-cause death (OR = 10.6017, p = 0.0035). In CABG patients, LVEF and the WMSI measured with early postoperative cardiac CT were comparable with echocardiography and predicted the development of MACE and all-cause death.

## Introduction

Coronary artery bypass grafting (CABG) restores myocardial contractile function in patients with multi-vessel coronary artery disease^1,2^, but this is not always effective^3,4^. Global ischemia and superimposed reperfusion injury might have deleterious effects on the heart^5–7^. Moreover, the time course of functional recovery after CABG varies from immediately intra-operatively to 1 year postoperatively^7–11^. Therefore, monitoring cardiac function in patients who have undergone CABG is important to predict their clinical outcome. Early anatomical recognition of bypass graft failure is important in terms of predicting clinical outcomes after CABG^12–15^. Computed tomography (CT) is a powerful noninvasive technique providing anatomical information on graft patency, with simultaneous evaluation of cardiac function without any need for additional examination^16^. Several studies have shown that CT is comparable to echocardiography and MRI for assessment of global left ventricular (LV) function and regional wall motion^17,18^. Moreover, CT is quicker and easier to perform than MRI, which makes it suitable for the examination of patients who have recently undergone CABG.

This study evaluated global and regional LV function using cardiac CT immediately after CABG and compared these with echocardiographic findings. In addition, we investigated the prognostic findings for predicting clinical outcome such as death and major adverse cardiac events (MACE) in patients who had undergone CABG.

## Methods

### Patients

This study was approved by the institutional review board at Ajou University Hospital (Approval No. AJIRB-MED-MDB-16–291), and the ethics committee waived the need for informed consent considering its retrospective nature. All methods were performed in accordance with the relevant guidelines and regulations. Of the 205 patients who underwent CABG from March 2011 to December 2014, 136 who underwent early postoperative cardiac CT (within 30 days after CABG) were included in our study. There were 106 men and 30 women, with an average age of 60.6 ± 9.6 years (range, 37–81 years). The patient characteristics are listed in Table 1. Before the operation, coronary angiography and baseline echocardiography were performed. Coronary angiography confirmed that 23 patients had one-vessel disease, 30 patients had two-vessel disease, and 83 patients had three-vessel disease. The early postoperative echocardiography was performed on average 7.9 ± 2.9 days after CABG. Because our study was retrospective in design, the interval between CABG and the follow-up echocardiography varied. Long-term follow-up echocardiography was defined as echocardiography that had been performed most recently 1 year ± 6 months following CABG.

**Table 1:** Patient clinical characteristics (n = 136).

Characteristics	Value
Age (yrs)	60.6 ± 9.6
Sex (Male/Female)	106/30
Blood pressure (systolic/diastolic)	123.0 ± 14.0/75.2 ± 10.1
BMI (m^2^/kg)	24.2 ± 3.1
Heart rate (bpm)	70.4 ± 9.1
Radiation dose (mSv)	11.3 ± 5.0
Interval (Baseline echo – CABG., days)	12.5 ± 30.2
Interval (ICA – CABG., days)	9.8 ± 21.6
Interval (CABG – early post-op. Echo, days)	7.9 ± 2.9
Interval (CABG – CT, days)	11.2 ± 5.2
Interval (early post-op. Echo – CT, days)	3.3 ± 5.0
Interval (CABG – long-term F/U Echo, days)	422.6 ± 127.4

BMI = body mass index, Echo = echocardiography, CABG = coronary artery bypass graft, ICA = invasive coronary angiography, F/U = follow-up.

### Echocardiography

Transthoracic echocardiography was performed by an experienced cardiologist, using the Philips iE33 (Philips Medical Systems, Andover, MA, USA) or GE Vivid 7 (GE-Vingmed Ultrasound, Horten, Norway) ultrasound system^19^. Echocardiographic examinations were evaluated by an attending cardiologist with 14 years of experience in echocardiography. The biplane Simpson method, using standard four- and two-chamber apical views derived the LV ejection fraction (LVEF). We defined LV systolic dysfunction as follows: mild 40–49%, moderate 30–39%, and severe ≤ 29%. Improvement in LVEF was defined as a ≥ 10% increase^20^. Regional wall motion abnormality (RWMA) was evaluated using a 16-segment model, which was divided into six basal segments, six middle segments, and four apical segments, as recommended by the American Society of Echocardiography^21^. By visual analyses of systolic wall thickening and wall motion, segments were assigned a wall motion score as follows: 1 = normal or hyperkinetic, 2 = hypokinetic, 3 = akinetic, and 4 = dyskinetic. The wall motion score index (WMSI) was calculated by dividing the sum of all wall motion scores by the total number of segments analyzed^6,22^. All 136 patients underwent both early postoperative echocardiography and cardiac CT; however, only 130 and 95 patients underwent baseline and long-term follow-up echocardiography, respectively.

### Coronary artery bypass grafting

CABG is schedules after careful evaluation of the clinical features, invasive coronary angiography findings, echocardiography results, and the patient’s condition. Our institution followed the current guidelines^23^ in terms of considering CABG for patients with symptomatic two- or three-vessel disease or high-grade left main stem coronary artery stenosis, and for patients with significant stenosis (>70%) of the proximal left anterior descending (LAD) artery, with one- or two-vessel disease including the proximal LAD artery. Tables 2 and 3 list the operations undergone by the patients included in this study. CABG was performed with cardiopulmonary bypass as follows. First, the left radial artery or saphenous vein graft (SVG) was harvested using an ultrasonic scalpel and retained in a blood-heparin solution. Standard median sternotomy was performed, and then the left internal mammary artery (LIMA) was removed. If it needed, a Y-graft was made by anastomosis between the LIMA and radial artery in an end to side fashion. After target portion stabilization of LAD anastomosis, LAD coronary arteriotomy, and end to side anastomosis between the LIMA and the LAD were performed. Anastomosis between the aorta and the harvested SVG was performed using a heartstring device. Following the Y-graft at the distal portion of the SVG, which was connected to the aorta, anastomosis with the target vessel was performed. To prevent kinking of the SVG, the position was fixed in place using glue. Finally, a total of 332 bypass grafts in 136 patients were performed.

**Table 2:** Operation methods.

Operation methods	Cases
LIMA to LAD (LIMA – LAD)	117
LIMA with Y-shape graft	10
LIMA to PDA (LIMA – PDA)	1
Aorta to LAD (Ao – LAD)	6
Aorta to diagonal branch (Ao – D)	76
Aorta to Ramus intermedius (Ao – RM)	13
Aorta to obtuse marginal branch (Ao – OM)	83
Aorta to RCA (Ao – RCA)	9
Aorta to PDA (Ao – PDA)	76
Aorta to posterolateral branch (Ao – PL)	23

LIMA = left internal mammary artery, LAD = left anterior descending, Ao = aorta, D = diagonal branch, OM = obtuse marginal branch, RCA = right coronary artery, PDA = posterior descending artery, RM = ramus intermedius, PL = posterolateral branch.

**Table 3:** Classification of operation methods according to vascular territory.

Operation methods	Cases
LAD territory only	20
LCX territory only	1
RCA territory only	2
LAD + LCX territory	17
LAD + RCA territory	13
LAD + LCX + RCA territory	83

LAD = left anterior descending, LCX = left circumflex, RCA = right coronary artery.

### Coronary CT angiography

**Figure 1:**
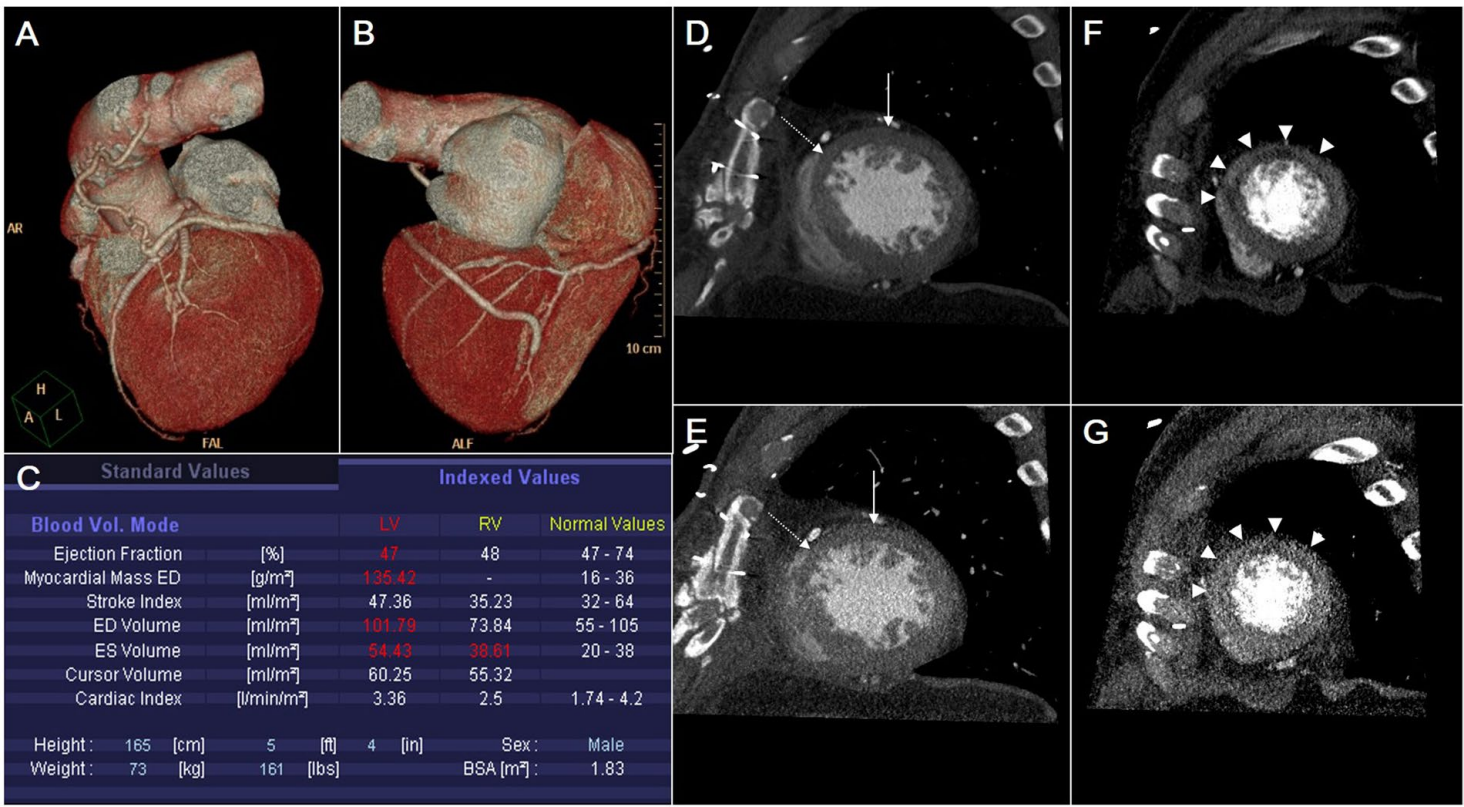
Functional analyses of the left ventricle (LV) using cardiac CT. (**A**) The patient underwent coronary artery bypass grafting (CABG). The left internal mammary artery (LIMA) was connected to the left anterior descending (LAD) artery. (**B**) Y-shaped graft using the saphenous vein connected to the posterior descending artery (PDA). (**C**) The ejection fraction of LV (LVEF) was 47%, representing mild systolic dysfunction. The LV chamber was enlarged to 102 mL/m^2^. The diastolic (**D**) and systolic (**E**) short-axis views of the mid-ventricular level show hypokinesia of segments 7 (arrow) and 8 (dotted arrow) of the LV myocardium. The diastolic (**F**) and systolic (**G**) short-axis views at the apical level show akinesia and subendocardial perfusion defects (arrowheads) at segments 13 and 14 of the LV myocardium. The wall motion score index (WMSI), measured using cardiac CT was 1.63, while the LVEF and WMSI, measured using early postoperative echocardiography, were 40% and 1.56, respectively. In the long-term follow-up echocardiography, the LVEF was improved to 68%, while the WMSI was aggravated to 1.62. The patient died after 4.7 year due to myocardial infarction.

Cardiac CT examinations were performed using a machine and protocol that we employed previously^19,24^. A prospective ECG tube current modulation technique were used. Contrast medium was injected using a split-bolus technique based on the patient’s body weight. First, 60 to 80 mL of pure, undiluted iodinated contrast material (Iomeron 350; Bracco SPA) was administered intravenously at 4.5 mL/s. Then, 40 mL of a 60%-to-40% mixture of contrast medium and saline was administered. A single observer with 15 years of experience in cardiac CT assessed cardiac CT images. To evaluate the bypassed arteries, images at 75% R–R intervals were used primarily for image reconstruction. If motion artifacts compromised diagnostic image quality, additional cardiac phases were reconstructed. Volume rendering and curved multiplanar reformation were routinely constructed using a commercial workstation (EBW, Philips Medical Systems). The LVEF was measured using an automated LV-endocardial and epicardial contour detection technique based on threshold-based blood volumes (Syngo.via imaging software, Siemens Healthcare, Cary, NC, USA) (Fig. 1). Papillary muscle was excluded from the LV chamber volume. RWMA was qualitatively evaluated using cine images (Syngo.via, Siemens Healthcare). Both LV systolic dysfunction and the WMSI were defined using the same method as echocardiographic analyses. Myocardial attenuation and wall thinning were also assessed. Subendocardial hypoperfusion was defined as hypoenhancement <50% of myocardial wall thickness, while transmural hypoperfusion was defined as hypoperfusion ≥ 50% of myocardial thickness. A myocardial perfusion score was assigned as follows: 1 = normal attenuation, 2 = subendocardial hypoperfusion, and 3 = transmural hypoperfusion. A myocardial perfusion score index (MPSI) was calculated by dividing the sum of all myocardial perfusion scores by the total number of segments analyzed. Myocardial wall thinning was defined as diastolic myocardial wall thickness <6 mm. The wall thinning segment number (WTSN) was the sum of the segments showing myocardial wall thinning.

### Clinical follow-up

The primary endpoint was all-cause mortality. The secondary endpoint was MACE, which included nonfatal myocardial infarction, nonfatal stroke, admission for unstable angina or heart failure, incident target vessel revascularization during follow-up, or death. Information on survival status and the development of MACE was collected between March 2011 and January 2019. Data gathering was performed using analyses of electronic charts for the follow-up clinical data in our hospital. The mean follow-up period was 5.9 ± 1.1 years.

### Statistical analyses

MedCalc (version 12.7.8; MedCalc Software, Mariakerke, Belgium) was used for all statistical analyses. Continuous variables were presented as the mean ± standard deviation (SD) or the median and compared by Wilcoxon test for paired samples. We used the Fisher exact test for comparisons of categorical variables. Correlation and Bland-Altman analyses were used to compare the LVEF between cardiac CT and echocardiography. The correlation coefficient was interpreted as follows: weak, ≤0.30; moderate, 0.41–0.60; strong, 0.61–0.80; and very strong, 0.81–1.00^25^. Limits of agreement (1.96 SD) on Bland-Altman analyses were calculated. Agreement for RWMA between the echocardiography and cardiac CT were calculated using weighted kappa statistics. The kappa value for agreement was interpreted as follows: poor, <0.20; fair, 0.21–0.40; moderate, 0.41–0.60; good, 0.61–0.80; excellent, 0.81–1.00^26^. Univariate logistic regression analyses were applied to investigate the prognostic value of the echocardiographic and cardiac CT findings for all-cause death and MACE. A value of p < 0.05 was considered statistically significant.

## Results

Early postoperative cardiac CT detected 10 (3.0%) graft failures in 10 patients (7.4%): 6 LIMA graft failures, 1 radial artery graft failure, and 4 SVG graft failures. Of the six patients who underwent coronary angiography, five were confirmed as graft failures.

**Figure 2:**
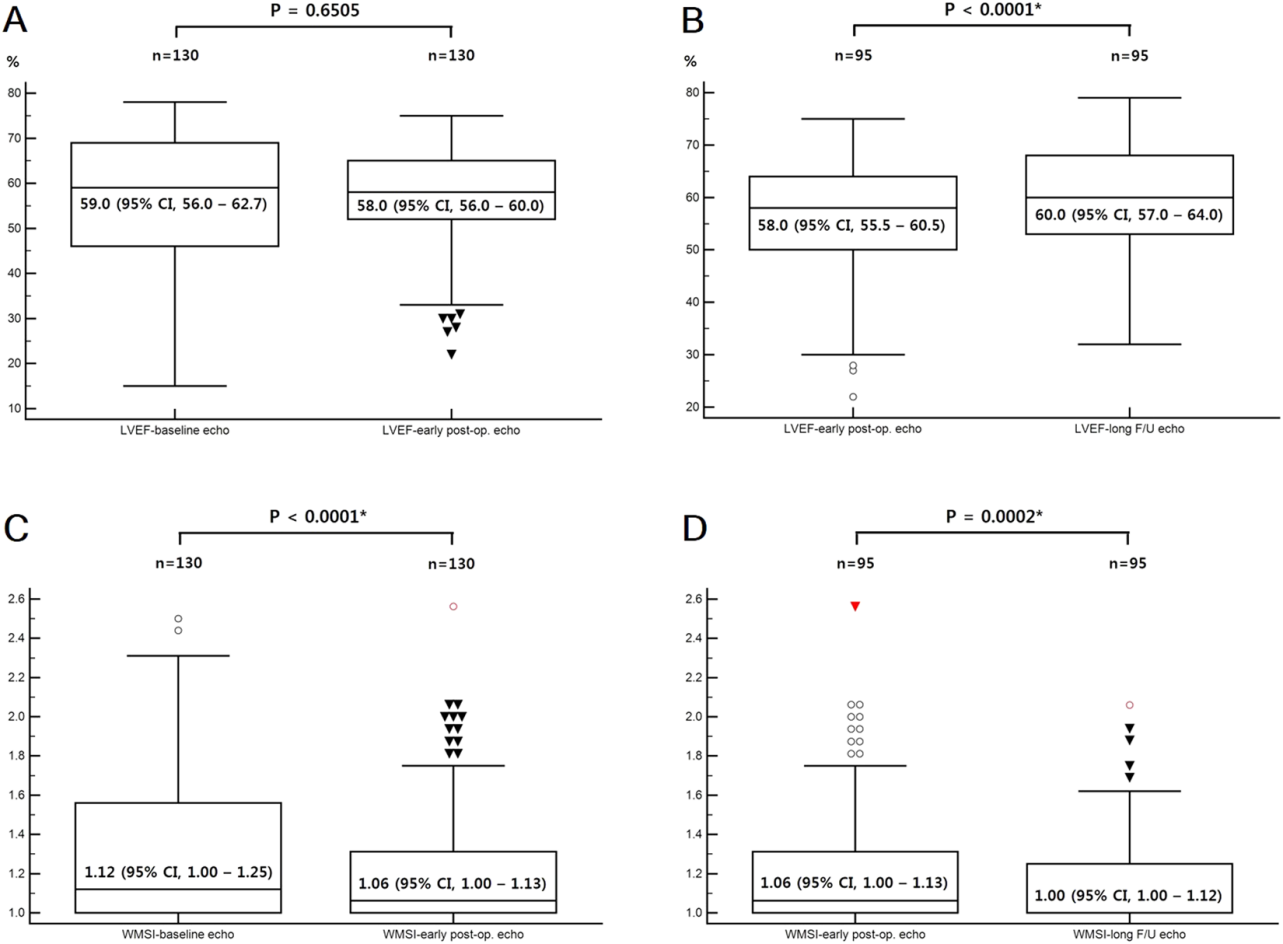
According to the Wilcoxon test, the LVEF was not significantly (p = 0.6505) increased in the early postoperative echocardiography (**A**) but was significantly increased (p < 0.0001) in the long-term follow-up echocardiography (**B**), whereas the WMSI was continuously decreased in the early postoperative- (p < 0.0001, **C)** and long-term follow-up echocardiography (p = 0.0002, **D**).

On baseline echocardiography, 29.2% (38/130) of the patients had LV systolic dysfunction, which decreased to 22.8% (31/136) in the early postoperative echocardiography and 12.6% (12/95) in the long-term follow-up echocardiography. Improvements in early postoperative echocardiography compared to baseline echocardiography were observed in 36.8% (14/38) of patients with LV systolic dysfunction, and in 65.8% (48/73) of patients with RWMA. A total of 65.4% (17/26) of patients with LV systolic dysfunction and 74.0% (37/50) of patients with RWMA in baseline echocardiography showed improvements in the long-term follow-up echocardiography. The Wilcoxon test revealed no significant increase in the LVEF (p = 0.6505) in early postoperative echocardiography, but this significantly increased (p < 0.0001) in the long-term follow-up echocardiography (Fig. 2). Meanwhile, the WMSI continuously decreased in early postoperative- (p < 0.0001) and long-term echocardiography (p = 0.0002).

**Figure 3:**
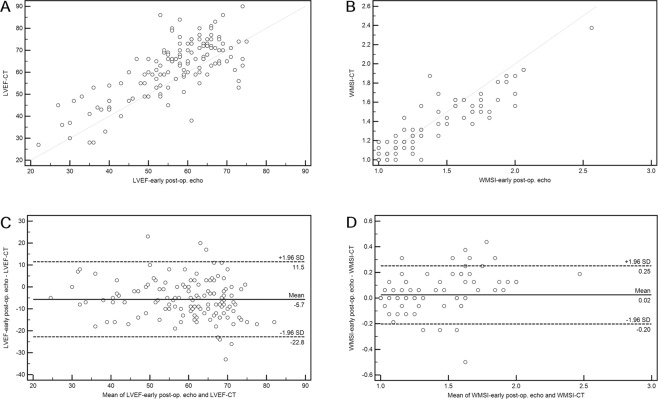
Correlation and agreement between the early postoperative echocardiography and the cardiac CT. Linear regression analyses show a strong correlation (r = 0.7500, p < 0.0001) for LVEF (**A**) and a very strong correlation (r = 0.9382, p < 0.0001) for WMSI (**B**). Bland-Altman analyses of the differences for LVEF and WMSI measured using early postoperative echocardiography and cardiac CT compared to the average for both methods. The mean differences were −5.7 ± 17.2% (limits of agreement = ±33.7%) for LVEF (**C**) and 0.02 ± 0.23 (limits of agreement = ±0.45) for WMSI (**D**).

A cardiac CT showed LV systolic dysfunction in 27 patients (19.9%, mild in 19 patients, moderate in 5 patients, and severe in 3 patients). The LVEF measured via the cardiac CT was significantly higher (56.2 ± 11.5% vs. 61.9 ± 12.9%, p = 0.0002) compared to those for early postoperative echocardiography, but the WMSI showed no significant difference (1.23 ± 0.33 vs. 1.21 ± 0.28, P = 0.5041) between the two methods. The LVEF and WMSI measured using cardiac CT correlated well with early postoperative echocardiographic assessment (r = 0.7500, p < 0.0001 and r = 0.9382, p < 0.0001, respectively) (Fig. 3). Bland-Altman analyses of the differences in LVEF and WMSI measured using early postoperative echocardiography and cardiac CT were comparable with the average of both methods. The mean differences were −5.7 ± 17.2% (limits of agreement = ±33.7%) for the LVEF and 0.02 ± 0.23 (limits of agreement = ±0.45) for the WMSI (Fig. 3).

RWMA was present in 336 segments (15.4%) in 70 patients (51.5%) with early postoperative echocardiography, and in 318 segments (14.6%) in 70 patients (51.5%) with cardiac CT (Table 4). Agreement of the RWMA between the two methods calculated by kappa statistics was excellent (k = 0.826; 95% CI, 0.796–0.856). Furthermore, a postoperative cardiac CT showed myocardial hypoenhancement in 177 segments (8.1%, 132 segments for subendocardial hypoperfusion and 45 segments for transmural hypoperfusion) in 77 patients (56.6%), and myocardial thinning in 65 segments (3.0%) in 27 patients (19.9%). The MPSI was not a predictor of improvement in the LVEF and WMSI, but WTSN was a negative predictor (odds ratio [OR] = 0.4031, p = 0.0060) of LVEF improvement in long-term echocardiography.

**Table 4:** Agreement in RWMA between echocardiography and cardiac CT.

Cardiac CT	Early post-op. echo				
	Normal	Hypokinesia	Akinesia	Dyskinesia	Total
Normal	1811	36	11	0	1858
Hypokinesia	20	124	42	0	186
Akinesia	9	8	114	0	131
Dyskinesia	0	0	0	1	1
Total	1840	168	167	1	2176

RWMA = regional wall motion abnormality.

During the follow-up period (mean, 5.9 ± 1.1 years), 17 patients (12.5%) died, and 40 patients (29.4%) developed MACE (Table 5). The mean survival period of those patients who died was 2.7 ± 2.1 years, and the mean event-free period of patients who developed MACE was 2.4 ± 1.9 years. Age, LVEF as measured using baseline echocardiography, the WMSI measured using baseline and long-term follow-up echocardiography, and cardiac CT significantly differed (p = 0.003–0.0489) between survival and death (Table 6). On the other hand, age and all of the functional parameters evaluated by echocardiography and cardiac CT significantly differed (p = 0.0001–0.0477) between patients with and without MACE (Table 6). Both the LVEF and WMSI measured using early postoperative echocardiography (OR = 0.9630, p = 0.0202, and OR = 5.0171, p = 0.0039, respectively) and cardiac CT (OR = 0.9625, p = 0.0091 and OR = 14.3605, p = 0.0001, respectively) predicted MACE, but only the WMSI measured using cardiac CT predicted all-cause death (OR = 10.6017, p = 0.0035) (Table 7).

**Table 5:** Patient outcomes.

Outcome	CABG (n = 136)
Primary endpoint (all cause death)	17 (12.5%)
Secondary endpoint (MACE)	40 (29.4%)
Nonfatal myocardial infarction	18 (11.8%)
Nonfatal stroke	6 (4.4%)*
Cardiovascular death	13 (9.6%)
Admission for unstable angina or heart failure	3 (2.2%)
Incident target vessel revascularization	7 (5.1%)*

MACE = major adverse cardiac events, *included overlapped events.

**Table 6:** Clinical and functional values according to all-cause death and MACE.

Value	All-cause death (95% CI)			MACE (95% CI)		
	Survival	Death	P-value	No	Developed	P-value
Age	59.0 (57.0–61.7)	72 (62.0–76.0)	0.0003*	59.5 (57.0–62.0)	63 (59.3–66.7)	0.0477*
Sex (M/F)	93/26	13/4	1.0000	73/23	33/7	0.4995
BMI	24.1 (23.4–24.7)	23.3 (21.7–25.3)	0.2210	24.3 (23.8–24.8)	23.7 (22.3–24.7)	0.3847
Graft failure	8	2	0.6132	4	6	0.0637
LVEF-baseline echo	60.0 (56.1–63.0)	49.0 (40.6–61.5)	0.0398*	62.5 (58.0–67.0)	53.5 (45.0–57.5)	0.0012*
LVEF-early post-op. echo	58.0 (56.0–61.0)	57.0 (43.0–64.9)	0.2774	59.0 (56.9–62.1)	55.5 (49.3–59.0)	0.0345*
LVEF-long-term F/U echo	61.0 (57.0–64.1)	55.5 (43.3–68.6)	0.1958	64.0 (60.0–67.0)	55.0 (50.0–57.9)	0.0013*
LVEF-CT	66.0 (63.0–66.7)	59.0 (51.0–64.0)	0.0597	66.0 (64.0–67.1)	57.5 (51.7–63.0)	0.0045*
WMSI-baseline echo	1.12 (1.00–1.25)	1.44 (1.12–1.65)	0.0489*	1.00 (1.00–1.19)	1.44 (1.22–1.62)	0.0008*
WMSI-early post-op. echo	1.06 (1.00–1.13)	1.25 (1.00–1.56)	0.1863	1.00 (1.00–1.06)	1.25 (1.08–1.44)	0.0024*
WMSI-long-term F/U echo	1.00 (1.00–1.06)	1.16 (1.05–1.68)	0.0260*	1.00 (1.00–1.00)	1.25 (1.12–1.42)	0.0001*
WMSI-CT	1.00 (1.00–1.13)	1.44 (1.00–1.63)	0.0259*	1.00 (1.00–1.06)	1.28 (1.08–1.54)	0.0006*
MPSI-CT	1.06 (1.00–1.06)	1.06 (1.00–1.19)	0.4933	1.06 (1.00–1.06)	1.06 (1.02–1.17)	0.0697
WTSN-CT	0.00 (0.00–0.00)	0.00 (0.00–0.00)	0.6547	0.00 (0.00–0.00)	0.00 (0.00–0.00)	0.2394

MACE = major adverse cardiac events, WMSI = wall motion score index, MPSI = myocardial perfusion score index, WTSN = wall thinning segment number, CI = confidence interval, *P < 0.05.

**Table 7:** Odds ratio to predict all-cause death and MACE (univariate analysis).

	All-cause death			MACE		
	OR	95% CI	P-value	OR	95% CI	P-value
Age	1.1301	1.0538–1.2119	0.0001*	1.0471	1.0052–1.0909	0.0230*
LVEF-baseline echo	0.9703	0.9392–1.0023	0.0722	0.9647	0.9405–0.9896	0.0048*
LVEF-early post-op. echo	0.9676	0.9283–1.0086	0.1253	0.9630	0.9325–0.9945	0.0202*
LVEF-long-term F/U echo	0.9470	0.8839–1.0146	0.1193	0.9214	0.8758–0.9695	0.0006*
LVEF-CT	0.9685	0.9327–1.0056	0.0982	0.9625	0.9347–0.9912	0.0091*
WMSI-baseline echo	2.6957	0.8566–8.4839	0.0992	3.3369	1.3439–8.2858	0.0089*
WMSI-early post-op. echo	3.7989	0.9939–14.5208	0.0588	5.0171	1.6397–15.3514	0.0039*
WMSI-long-term F/U echo	10.0649	0.9476–106.8997	0.0693	29.3008	3.7224–230.6403	0.0005*
WMSI-CT	10.6017	2.1737–51.7063	0.0035*	14.3605	3.5894–57.4534	0.0001*

MACE = major adverse cardiac events, WMSI = wall motion score index, CI = confidence interval, *P < 0.05.

## Discussion

Cardiac CT evaluation of graft patency is appropriate for symptomatic patients who undergo CABG, but not for asymptomatic patients who underwent CABG within the past 5 years^27^. However, it remains unclear whether immediate postoperative cardiac CT after CABG is useful. In practice, most surgeons require that surgical outcomes are monitored prior to patient discharge, because the quality of the surgical anastomosis and graft patency influence long-term outcomes^12,13^. Cardiac CT can accurately provide anatomic information about graft patency^14^. Early graft failure after CABG is detected in 4.6–10.3% of grafts^14,28,29^. Our CABG results were excellent compared to previous reports; graft failure was detected in only 3.0% of the total grafts and only 7.4% of the study population. Our result was similar to the in-hospital acute graft failure rate (3.4% of graft and 6.8% of patients) reported by Arampatzis *et al*.^30^.

Besides graft patency evaluation, cardiac CT allows simultaneous evaluation of cardiac function without the need for additional examinations. We suggest that LV function evaluated by cardiac CT is useful for predicting the long-term outcomes of patients who have undergone CABG. Both the LVEF and WMSI measured using early postoperative cardiac CT (OR = 0.9625 and OR = 14.3605, respectively) predicted MACE, and also the WMSI measured using cardiac CT predicted all-cause death (OR = 10.6017). Several predictors such as age, female sex, and low ejection fraction are associated with long-term outcomes, including death and the development of cardiac events after CABG^31–36^. However, to the best of our knowledge, regional wall motion evaluated with cardiac CT have not been investigated as a means to predict death and MACE in patients who have undergone CABG.

A recent meta-analysis showed that CT assessment of global LV function complemented echocardiographic and MRI data^37^. Cury *et al*.^16^ and Henneman *et al*.^38^ reported strong (r = 0.68) and very strong (r = 0.91) correlations, respectively, between CT and echocardiography to evaluate LVEF. In our study, assessment of LVEF using CT showed a strong correlation (r = 0.7500) with echocardiography. However, LVEF was slightly overestimated by cardiac CT with a mean difference of −5.7 ± 17.2% compared to echocardiography. The differences observed for the LVEF in our study between echocardiography and cardiac CT may have arisen from this mismatch of inclusion or exclusion of the papillary muscle, depending on the imaging modalities. The agreement between CT and echocardiography for assessing regional wall motion can be variable (kappa value = 0.61–0.82)^16,38–41^. In our study, the overall agreement between CT and echocardiography for regional wall motion was very strong (k = 0.826). Therefore CT may be used as an alternative noninvasive method to estimate regional wall motion in patients with poor acoustic windows for echocardiography or patients with contraindications to MRI. During the early postoperative period after CABG, patients experience many problems such as respiratory difficulties, and a rapid, irregular heartbeat. In these cases, cardiac CT has the advantage of a relatively shorter acquisition time and superior clinical availability for use in postoperative patients^15,16^.

Myocardial perfusion abnormality is another prognostic factor for ischemic heart disease^42,43^, but the effects of myocardial perfusion abnormality on cardiac CT for predicting post-CABG outcomes have not yet been evaluated. In our study, the MPSI was not a predictor for functional improvement of LVEF or regional wall motion. We presumed that the prognostic effect could be counteracted by the presence of global stunning due to perioperative ischemia that occurs during CABG or following reperfusion injury after CABG^5–7^. We also evaluated myocardial thickness as another predictor of contractile functional recovery. Myocardial wall thinning is pathologically associated with myocardial necrosis, and is considered a marker of chronic myocardial infarction^44^. As a result in our study, WTSN was a negative predictor (OR = 0.4031) of LVEF improvement. In addition to LV function improvement, right ventricular (RV) function also improves after CABG, especially in patients with low baseline RV ejection fraction^45^. Preoperative RV systolic dysfunction predicts long-term cardiac re-hospitalization and cardiovascular death^46,47^. Preoperative left atrial enlargement (left atrial diameter ≥ 4 cm) is a strong independent predictor of mortality after CABG^48^. Thus, more studies employing cardiac CT are required to reveal the increasing prognostic utilities of RV function and left atrial diameter in such patients.

### Study limitations

There were several limitations to our study. First, the number of subjects was relatively small, so interpretation of statistical significance and subgroup analyses may have some restrictions. Second, this study was designed as a single-center and was retrospective in nature, performed using reviewing medical records, which could have resulted in a certain degree of selection bias. Third, serial follow-up echocardiography was not performed in all patients, and there was a large time interval range between CABG and follow-up echocardiography due to the retrospective nature of the study. Fourth, the visual definition of regional wall motion is subjective and operator-dependent, which means that overestimation or underestimation of RWMA was a possibility. However, assessment of the reproducibility of regional wall motion abnormalities was difficult. Because our study was based on retrospectively reviewing cardiac CT reading paper that only one observer assessed cardiac CT images. As the LVEF was measured automatically, reproducibility was not a concern. Finally, we used a retrospective ECG-triggering protocol to enable cardiac functional analyses. Moreover, CT examination of bypass patients necessitates an increased scan length for the z-axis, which results in a higher radiation dose. Therefore, we applied tube current modulation to reduce the maximum tube current to 20%, and a reduced tube voltage^49^. Nevertheless, the effective radiation dose in our study population was 11.3 ± 5.0 mSv. The maximal tube current modulation technique with a dual-source CT scanner can reduce tube current to as low as 4% outside a predefined window. The use of an iterative reconstruction algorithm to reduce image noise is another method to reduce radiation dose while maintaining diagnostic image quality^50^.

## Conclusions

Our patients showed improved global LV systolic function and regional wall motion after CABG. The LVEF and WMSI, measured using early postoperative cardiac CT, were comparable with echocardiography and predicted the development of MACE and all-cause death in patients who had undergone CABG. Thus, cardiac CT is a viable alternative to echocardiography when use of the latter may be difficult.

## References

[1] Wolf NM (1978). Left ventricular function following coronary bypass surgery. Circulation.

[2] Lorusso R (2001). Long-term results of coronary artery bypass grafting procedure in the presence of left ventricular dysfunction and hibernating myocardium. Eur. J. Cardiothorac. Surg..

[3] Kato M, Nakashima Y, Levine J, Goldiner PL, Oka Y (1993). Does transesophageal echocardiography improve postoperative outcome in patients undergoing coronary artery bypass surgery?. J. Cardiothorac. Vasc. Anesth..

[4] Swaminathan M (2007). Deterioration of regional wall motion immediately after coronary artery bypass graft surgery is associated with long-term major adverse cardiac events. Anesthesiology.

[5] Kloner RA, Przyklenk K, Patel B (1989). Altered myocardial states: The stunned and hibernating myocardium. Am. J. Med..

[6] Søraas CL (2011). Echocardiographic demonstration of improved myocardial function early after coronary artery bypass graft surgery. Interact. Cardiovasc. Thorac. Surg..

[7] Mavi M (2005). Hemodynamic and transesophageal echocardiographic analysis of global and regional myocardial functions, before and immediately after coronary artery bypass surgery. J. Card. Surg..

[8] Vanoverschelde JL (2000). Time course of functional recovery after coronary artery bypass graft surgery in patients with chronic left ventricular ischemic dysfunction. Am. J. Cardiol..

[9] Knapp M (2007). Myocardial contractility improvement after coronary artery by-pass grafting in a 1-year observation: The role of myocardial viability assessment. Cardiol. J..

[10] Yee NP, Siu AM, Davis J, Kao J (2016). Recovery of Left Ventricular Function After Percutaneous Coronary Intervention Compared to Coronary Artery Bypass Grafting in Patients with Multi-Vessel Coronary Disease and Left Ventricular Dysfunction. Hawaii. J. Med. Public. Health.

[11] Hwang HY (2017). Cardiac Magnetic Resonance Predictor of Ventricular Function after Surgical Coronary Revascularization. J. Korean Med. Sci..

[12] Fitzgibbon GM (1996). Coronary bypass graft fate and patient outcome: angiographic follow-up of 5,065 grafts related to survival and reoperation in 1,388 patients during 25 years. J. Am. Coll. Cardiol..

[13] Halabi A (2005). Relation of early saphenous vein graft failure to outcomes following coronary artery bypass surgery. Am. J. Cardiol..

[14] Vernhet-Kovacsik H (2006). Early postoperative assessment of coronary artery bypass graft patency and anatomy: value of contrast-enhanced 16-MDCT with retrospectively ECG-gated reconstructions. AJR Am. J. Roentgenol..

[15] Dikkers R (2007). The benefit of 64-MDCT prior to invasive coronary angiography in symptomatic post-CABG patients. Int. J. Cardiovasc. Imaging.

[16] Cury RC (2008). Comprehensive assessment of myocardial perfusion defects, regional wall motion, and left ventricular function by using 64-section multidetector CT. Radiology.

[17] Juergens KU, Fischbach R (2006). Left ventricular function studied with MDCT. Eur. Radiol..

[18] Orakzai SH, Orakzai RH, Nasir K, budoff MJ (2006). Assessment of cardiac function using Multidetector row computed tomography. J. Comput. Assis Tomogr..

[19] Cho JY, Sun JS, Sur YK, Park JS, Kang DK (2015). Relationship between left ventricular mass and coronary artery disease in young adults: a single-center study using cardiac computed tomography. Int. J. Cardiovasc. Imaging Suppl..

[20] Adachi Y (2016). Determinants of Left Ventricular Systolic Function Improvement Following Coronary Artery Revascularization in Heart Failure Patients With Reduced Ejection Fraction (HFrEF). Int. Heart J..

[21] Schiller NB (1989). Recommendations for quantitation of the left ventricle by two dimensional echocardiography. American Society of Echocardiography Committee on Standards, Subcommittee on Quanititation of Two-Dimensional Echocardiograms. J. Am. Soc. Echocardiogr..

[22] Awan (2007). H. Early effects of coronary artery bypass grafting on left ventricular regional wall motion abnormalities. J. Coll. Physicians Surg. Pak..

[23] JCS Joint Working Group. (2013). Guidelines for elective percutaneous coronary intervention in patients with stable coronary artery disease (JCS 2011) published in 2012–digest version. Circ. J..

[24] You S, Sun JS, Park SY, Baek Y, Kang DK (2016). Relationship between indexed epicardial fat volume and coronary plaque volume assessed by cardiac multidetector CT. Medicine.

[25] Akoglu H (2018). User’s guide to correlation coefficients. Turk. J. Emerg. Med..

[26] Altman D. G. Practical statistics for medical research. Chapman and Hall, London, pp 404 (1991).

[27] Taylor AJ (2010). American College of Cardiology Foundation Appropriate Use Criteria Task Force; Society of Cardiovascular Computed Tomography; American College of Radiology; American Heart Association; American Society of Echocardiography; American Society of Nuclear Cardiology; North American Society for Cardiovascular Imaging; Society for Cardiovascular Angiography and Interventions; Society for Cardiovascular Magnetic Resonance, Kramer CM, Berman D, Brown A, Chaudhry FA, Cury RC, Desai MY, Einstein AJ, Gomes AS, Harrington R, Hoffmann U, Khare R, Lesser J, McGann C, Rosenberg A, Schwartz R, Shelton M, Smetana GW, Smith SC Jr. ACCF/SCCT/ACR/AHA/ASE/ASNC/NASCI/SCAI/SCMR 2010 appropriate use criteria for cardiac computed tomography. A report of the American College of Cardiology Foundation Appropriate Use Criteria Task Force, the Society of Cardiovascular Computed Tomography, the American College of Radiology, the American Heart Association, the American Society of Echocardiography, the American Society of Nuclear Cardiology, the North American Society for Cardiovascular Imaging, the Society for Cardiovascular Angiography and Interventions, and the Society for Cardiovascular Magnetic Resonance. J. Am. Coll. Cardiol..

[28] Berger PB, Alderman EL, Nadel A, Schaff H (1999). Frequency of early occlusion and stenosis in a left internal mammary artery to left anterior descending artery bypass graft after surgery through a median sternotomy on conventional bypass. Circulation.

[29] Jeong YM (2009). Evaluation of Coronary Artery Bypass Grafts in the Early Postoperative Period Using 64-Slice MDCT. J. Korean Soc. Radiol..

[30] Arampatzis CA (2016). Graft failure prior to discharge after coronary artery bypass surgery: a prospective single-centre study using dual 64-slice computed tomography. EuroIntervention.

[31] Ahmed WA, Tully PJ, Knight JL, Baker RA (2011). Female sex as an independent predictor of morbidity and survival after isolated coronary artery bypass grafting. Ann. Thorac. Surg..

[32] Vickneson K (2019). Coronary artery bypass grafting in patients with low ejection fraction: what are the risk factors?. J. Cardiovasc. Surg..

[33] Fortescue EB, Kahn K, Bates DW (2001). Development and validation of a clinical prediction rule for major adverse outcomes in coronary bypass grafting. Am. J. Cardiol..

[34] van Straten AH (2010). Effect of body mass index on early and late mortality after coronary artery bypass grafting. Ann. Thorac. Surg..

[35] Nalysnyk L, Fahrbach K, Reynolds MW, Zhao SZ, Ross S (2003). Adverse events in coronary artery bypass graft (CABG) trials: a systematic review and analysis. Heart.

[36] Völzke H (2007). Outcome after coronary artery bypass graft surgery, coronary angioplasty and stenting. Int. J. Cardiol..

[37] van der Vleuten PA (2006). Quantification of global left ventricular function: comparison of multidetector computed tomography and magnetic resonance imaging. A meta-analysis and review of the current literature. Acta Radiol..

[38] Henneman MM (2006). Assessment of global and regional left ventricular function and volumes with 64-slice MSCT: a comparison with 2D echocardiography. J. Nucl. Cardiol..

[39] Ko SM, Kim YJ, Park JH, Choi NM (2010). Assessment of left ventricular ejection fraction and regional wall motion with 64-slice multidetector CT: a comparison with two-dimensional transthoracic echocardiography. Br. J. Radiol..

[40] Fischbach R (2007). Assessment of regional left ventricular function with multidetector-row computed tomography versus magnetic resonance imaging. Eur. Radiol..

[41] Butler J (2007). Comparison of multidetector computed tomography and two-dimensional transthoracic echocardiography for left ventricular assessment in patients with heart failure. Am. J. Cardiol..

[42] Bezerra HG (2011). Incremental value of myocardial perfusion over regional left ventricular function and coronary stenosis by cardiac CT for the detection of acute coronary syndromes in high-risk patients: a subgroup analysis of the ROMICAT trial. J. Cardiovasc. Comput. Tomogr..

[43] Cho YH (2019). Reference parameters for left ventricular wall thickness, thickening, and motion in stress myocardial perfusion CT: Global and regional assessment. Clin. Imaging.

[44] Cwajg JM (2000). End-diastolic wall thickness as a predictor of recovery of function in myocardial hibernation: relation to rest-redistribution T1-201 tomography and dobutamine stress echocardiography. J. Am. Coll. Cardiol..

[45] Joshi SB (2010). Right ventricular function after coronary artery bypass graft surgery - a magnetic resonance imaging study. Cardiovasc. Revasc Med..

[46] Lella LK (2015). Reduced Right Ventricular Function Predicts Long-Term Cardiac Re-Hospitalization after Cardiac Surgery. PLoS One.

[47] Pouleur AC (2016). Right Ventricular Systolic Dysfunction Assessed by Cardiac Magnetic Resonance Is a Strong Predictor of Cardiovascular Death After Coronary Bypass Grafting. Ann. Thorac. Surg..

[48] Ibrahim KS, Mayyas FA, Kheirallah K, AlWaqfi NR, Van Wagoner DR (2017). Is Left Atrial Size a Predictor of Mortality after Coronary Artery Bypass Surgery? A Single Center Study. Acta Cardiol. Sin..

[49] Mahnken AH (2009). Left ventricular function can reliably be assessed from dual-source CT using ECG-gated tube current modulation. Invest. Radiol..

[50] Fuchs TA (2014). Coronary computed tomography angiography with model-based iterative reconstruction using a radiation exposure similar to chest X-ray examination. Eur. Heart J..

